# Evaluating dp-ucMGP, tMGP, and Fetuin-A as Markers of Combined Arterial Atherosclerotic Disease: A Cross-Sectional Study

**DOI:** 10.3390/ijms27209391

**Published:** 2026-10-21

**Authors:** Teodora Bianca Tirla-Sirca, Mariana Eugenia Mureșan, Luciana Dobjanschi, Tamás Ilyés, Rodica Anamaria Negrean, Laura Grațiela Vicaș, Corina Cinezan, Elisabeta Cristina Crăciun (Rusu), Camelia Mraz, Alexandra M. Crăciun

**Affiliations:** 1 Doctoral School of Biomedical Sciences, University of Oradea, No. 1 University Street, 410087 Oradea, Romania; sirca.teodorabianca@student.uoradea.ro (T.B.T.-S.); lvicas@uoradea.ro (L.G.V.); craciun.elisabetacristina@student.uoradea.ro (E.C.C.); 2 Department of Preclinical Disciplines, Faculty of Medicine and Pharmacy, University of Oradea, 10 Piata 1 Decembrie Street, 410073 Oradea, Romania; rodicanegrean@uoradea.ro; 3 Department of Molecular Sciences, University of Medicine and Pharmacy “Iuliu Hațieganu”, 400012 Cluj-Napoca, Romania; tamas.ilyes@umfcluj.ro; 4 Department of Pharmacy, Faculty of Medicine and Pharmacy, University of Oradea, 29 Jiga Street, 410028 Oradea, Romania; 5 Department of Medical Disciplines, Faculty of Medicine and Pharmacy, University of Oradea, 410073 Oradea, Romania; ccinezan@uoradea.ro; 6 Laboratory Medicine, Bihor Public Health Directorate, Strada Libertății 34, 410040 Oradea, Romania; camelia.mraz@yahoo.com; 7 Medical Biochemistry, Department of Molecular Sciences, Faculty of Medicine, “Iuliu Hațieganu” University of Medicine and Pharmacy, 400349 Cluj-Napoca, Romania; acraciun@umfcluj.ro

**Keywords:** dp-ucMGP, matrix Gla protein, vascular calcification, atherosclerosis, fibrocalcific plaque, arterial stenosis, Fetuin-A, cardiovascular biomarkers

## Abstract

Vascular calcification and atherosclerotic arterial disease are complex processes involving multiple inhibitory and pro-calcific mechanisms. Matrix Gla protein (MGP) is an important inhibitor of vascular calcification, and its inactive dephosphorylated–uncarboxylated form (dp-ucMGP) has been proposed as a biomarker of cardiovascular risk. The present study aimed to evaluate the association of circulating dp-ucMGP, total MGP (tMGP), and Fetuin-A concentrations as markers for calcification associated with the presence of fibrocalcific plaques and/or arterial stenosis. A total of 160 patients were included in this observational study. Patients were classified according to the presence of fibrocalcific plaques and/or arterial stenosis. The study group included 136 patients, whereas 24 patients without fibrocalcific plaques or stenosis served as the control group. dp-ucMGP concentrations were significantly higher in the study group [1.29 vs. 1.07, *p* = 0.047]. In contrast, no significant differences were observed for tMGP [15.79 vs. 12.67, *p* = 0.532] or Fetuin-A [388.50 vs. 403.05, *p* = 0.894]. Glucose concentrations were significantly higher in the study group (*p* < 0.001), together with significant differences in fibrinogen, urea, creatinine, LDL cholesterol, and uric acid. Spearman correlation analysis showed weak positive correlations between dp-ucMGP and urea (ρ = 0.175, *p* = 0.034), creatinine (ρ = 0.183, *p* = 0.026), and triglycerides (ρ = 0.197, *p* = 0.017). Higher circulating dp-ucMGP concentrations were associated with the presence of fibrocalcific plaques and/or arterial stenosis, whereas tMGP and Fetuin-A did not differ significantly between groups. These findings support the potential relevance of dp-ucMGP as a biomarker associated with fibrocalcific and stenotic arterial disease. Further studies are required to determine its independent predictive value and clinical utility.

## 1. Introduction

Vascular calcification is defined as the accumulation of calcium and phosphate deposits in blood vessels, which reduces vessel elasticity. A variety of factors contribute to vascular calcification, which is prevalent in older adults. Still, it is found earlier in patients with dyslipidemia, arterial hypertension, smoking, and even chronic inflammation and mineral metabolism disorders. The underlying mechanism is the conversion of vascular smooth muscle cells into osteoblast-like cells, which are closely related to bone tissue in blood vessels. This process is facilitated by the loss of elastin and proteins such as matrix Gla protein (MGP) and Fetuin-A [1].

Calcified deposits within blood vessels are located in specific layers of the aortic wall and are associated with distinct disease types. Intimal calcification develops within atherosclerotic plaques and is associated with vascular smooth muscle cells and macrophages, whereas medial calcification—often referred to as “Monckeberg’s medial sclerosis”—affects the medial layer of the aortic wall and is associated with collagen and elastin fibers as well as vascular smooth muscle cells and leads to arterial stiffness, arterial hypertension, and left ventricular hypertrophy [2,3]. Medial calcification is typically associated with chronic kidney disease, although it is also linked to conditions such as diabetes mellitus, hypertension, advanced age, and osteoporosis. Notably, this form of calcification contributes to increased blood vessel rigidity, which elevates the risk of cardiovascular complications even in the absence of vascular lumen narrowing [4].

Matrix Gla protein (MGP), a 14 kDa protein with 84 amino acids, is a member of the vitamin K-dependent protein family. It is a significant inhibitor of arterial calcification and is also involved in bone metabolism and blood coagulation [1,5]. It is composed of five Glu residues and three serine residues, and its form is contingent upon carboxylation and phosphorylation [6]. The deficiency of vitamin K is indicative of vascular calcification, as evidenced by elevated levels of the inactive unphosphorylated and uncarboxylated form (dp-ucMGP). Only the active form of MGP (carboxylated) is responsible for protection against calcification by inhibition of bone morphogenic protein 2 (BMP-2), a protein responsible for inflammation and oxidative stress found in atherosclerotic sites [5]. The inactive form is used in studies for assessing vitamin K status, calcification state, and is even used for the determination of the cardiovascular risk [6]. A study on the correlation between dp-uc MGP and atherosclerosis in carotids and peripheral arteries showed that plaques from the peripheral lower limbs had a markedly greater calcification burden than carotid plaques, with larger calcified volumes and more calcified particles. Despite higher circulating dp-ucMGP levels in patients with carotid disease, no significant relationship was observed between dp-ucMGP concentrations and plaque calcification measures [7]. These findings support evidence that calcification is more extensive in peripheral arterial lesions than in carotid plaques [8]. The increased dp-ucMGP levels in carotid patients may be related to the frequent coexistence of peripheral arterial disease in this population. Overall, dp-ucMGP did not reflect the extent of vascular calcification in their cohort [7].

Fetuin-A, a glycoprotein from the cystatin family, is a 349-amino-acid compound, has two chains, one heavy and one light, and is distributed in extracellular fluids. It acts as a major systemic defense against ectopic mineral deposition by binding calcium and phosphate and promoting their incorporation into stable calciprotein particles, thereby limiting mineral precipitation and aggregation. Through these mechanisms, Fetuin-A plays a role in the prevention of vascular calcification, and it may modulate vascular smooth muscle cell response against pro-calcific stimuli [1,9,10,11]. Experimental studies have shown that the deficiency of Fetuin-A is responsible for extensive ectopic soft-tissue calcification, supporting its protective role against mineral deposition. In addition, Fetuin-A has anti-inflammatory properties and is considered a negative acute-phase protein [11]. Reduced circulating Fetuin-A concentration has also been associated with chronic inflammation in patients with end-stage renal disease, suggesting a role between Fetuin-A deficiency, inflammation, and vascular calcification [12].

## 2. Results

### 2.1. Overall Study Population

A total of 160 patients were included in the analysis (Table 1). Median age was 67 years (66.4+/−20.8), and 87 patients (54.4%) were female; 95 patients (59.4%) resided in an urban setting. The most prevalent comorbidities were arterial hypertension (88.1%), chronic heart disease (64.2%), and dyslipidemia-related conditions, alongside diabetes mellitus (33.1%) and chronic kidney disease (29.4%). Of the total cohort, 136 patients (85.0%) met the pre-specified definition of combined arterial atherosclerotic disease (FCP+), while 24 patients (15.0%) did not have fibrocalcific plaques or stenosis (FCP−). The study and control groups differed significantly in age (median 68 vs. 54 years, *p* < 0.001) The groups were comparable with respect to sex, place of residence, carotid or mitral stenosis, obesity, hepatic steatosis, stroke, diabetes mellitus, cholelithiasis, nephrolithiasis, varicose veins, venous insufficiency, or peripheral arterial stenosis (no significant differences). The groups were sex-matched with respect to place of residence, chronic ischemic heart disease, chronic kidney disease, obesity, hypertension, stroke, diabetes mellitus, aortic stenosis, varicose veins, and, in our group, stenosis and fibrocalcific plaques.

Median circulating biomarker levels in the overall cohort were 15.79 ng/mL for tMGP, 1.29 ng/mL for dp-ucMGP, and 388.5 µg/mL for Fetuin-A (Table 1).

### 2.2. Study Population and Clinical Characteristics

For the primary analysis, patients were classified according to the presence of combined arterial atherosclerotic disease, defined as the presence of fibrocalcific plaques and/or arterial stenosis involving the carotid and/or arterial limb territories. According to this classification, 136 patients (85.0%) were included in the study group (FCP+), whereas 24 patients (15.0%) had neither fibrocalcific plaques nor stenosis and were classified as the control group (FCP−).

Because continuous variables deviated from a normal distribution, results are presented as the median and interquartile range (Q1–Q3). Comparisons between the two independent groups were performed using the Mann–Whitney U test. Categorical variables are presented as absolute numbers and percentages, with the corresponding *p*-values shown. A two-sided *p*-value < 0.05 was considered statistically significant.

Patients with combined arterial atherosclerotic disease were substantially older than those without (median 68.00 vs. 54.00 years; *p* < 0.001). They also had higher glucose levels (103.00 vs. 91.50 mg/dL; *p* < 0.0001), uric acid (5.92 vs. 4.68 mg/dL; *p* < 0.0001), creatinine (0.93 vs. 0.77 mg/dL; *p* = 0.002), and urea (37.67 vs. 32.53 mg/dL; *p* = 0.019), consistent with a more metabolically and renally compromised profile in the FCP+ group. Fibrinogen was also modestly higher in the FCP+ group (374.40 vs. 330.00 mg/dL; *p* = 0.046).

A notable inverse pattern was observed for LDL cholesterol, which was lower in patients with combined arterial atherosclerotic disease (100.00 vs. 120.50 mg/dL; *p* = 0.048). This pattern is compatible with more extensive prior lipid-lowering treatment in patients already carrying an atherosclerotic diagnosis, though the present analysis cannot confirm treatment status. CRP approached but did not cross the significance threshold *p* value (2.90 vs. 1.80 mg/L; *p* = 0.052). No significant differences between groups were found for triglycerides, transaminases (GOT, GPT, GGT), bilirubin fractions, HDL cholesterol, or ESR (all *p* > 0.05) (Table 2).

Because several clinical variables had more than two categories (e.g., dyslipidemia: no dyslipidemia, untreated dyslipidemia, treated dyslipidemia), comparisons among groups were performed using the Kruskal–Wallis test, followed, when significant, by Dunn’s post hoc test with Bonferroni correction.

#### 2.2.1. Dyslipidemia

There were significant differences between the three dyslipidemia categories for age (*p* = 0.003), urea (*p* = 0.008), total cholesterol (*p* < 0.0001), LDL cholesterol (<0.0001), and HDL cholesterol (*p* = 0.017). Post hoc analysis with Bonferroni correction showed that patients receiving treatment for dyslipidemia were older than patients with untreated dyslipidemia (median age, 70 vs. 65 years; *p* = 0.003). Importantly, no significant differences were observed among the three dyslipidemia groups for tMGP, dp-ucMGP, or Fetuin-A (*p* > 0.05) (Table 3).

#### 2.2.2. Hyperuricemia

Urea (*p* < 0.0001), uric acid (*p* < 0.0001), creatinine (*p* < 0.0001), triglycerides (*p* < 0.0001), age (*p* = 0.005), total cholesterol (*p* = 0.028), CRP (*p* < 0.05), and LDL-C (*p* = 0.014) differed significantly across hyperuricemia subgroups. Fetuin-A approached but did not cross the significance threshold (*p* = 0.052), while tMGP (*p* = 0.854) and dp-ucMGP (0.517) showed no evidence of a group difference (Table 4).

#### 2.2.3. Post Hoc Dunn Test with Bonferroni Correction

##### Dyslipidemia Subgroups

For age, creatinine, and D-bilirubin, the significant omnibus effect was driven specifically by the comparison between the untreated dyslipidemia group and the under-treatment group (all adjusted *p* ≤ 0.05), with no significant difference between the other groups.

##### Hyperuricemia Subgroups

For age, urea, and triglycerides, the significant omnibus effect was driven by the comparison between the untreated hyperuricemia group and the group without hyperuricemia (all adjusted *p* ≤ 0.05), rather than by either group’s comparison with the under-treatment subgroup. Total cholesterol instead showed a significant pairwise difference between those with hyperuricemia without treatment and those under treatment (*p* < 0.05) and between those without hyperuricemia and those under treatment (*p* < 0.05).

### 2.3. Primary Biomarker Analysis: dp-ucMGP, tMGP, and Fetuin-A

The central hypothesis of this study concerned whether dp-ucMGP, tMGP, and Fetuin-A differed by combined arterial atherosclerotic disease status. Of the three biomarkers, only dp-ucMGP showed a statistically significant association: patients with combined arterial atherosclerotic disease had higher dp-ucMGP levels (median 1.29 ng/mL) than those without (median 1.07 ng/mL, *p* = 0.047).

tMGP was numerically higher in the disease group (median 15.79 vs. 12.67 ng/mL) but the difference did not reach statistical significance (*p* = 0.532). Fetuin-A showed essentially no separation between groups (median 388.50 vs. 403.05 µg/mL, *p* = 0.894).

The only MGP-related parameter that differed significantly between the two carotid and limb phenotypes was dp-ucMGP (Table 5).

### 2.4. Spearman Correlation Analysis

To characterize how the three biomarkers relate to continuous clinical and metabolic parameters, Spearman rank correlations were computed across the full cohort (Table 6).

**dp-ucMGP** correlated positively with markers of renal function—urea (ρ = 0.175, *p* = 0.034) and creatinine (ρ = 0.183, *p* = 0.026)—and with triglycerides (ρ = 0.197, *p* = 0.017). No correlation was found with age, lipid parameters, inflammatory markers (CRP, ESR), or liver enzymes.

**tMGP** correlated positively with fibrinogen (ρ = 0.208, *p* = 0.014), but with no other continuous variable tested, including renal function markers, lipids, or inflammatory indices.

**Fetuin-A** correlated positively with triglycerides (ρ = 0.176, *p* = 0.029); no other significant correlations were observed.

The moderate positive correlation between tMGP and dp-ucMGP (ρ = 0.443) indicates partial biological overlap between the two MGP species, while triglycerides emerged as the only clinical variable correlated with all three biomarkers, though the strength of that correlation was modest and consistent across markers (ρ = 0.176–0.197).

## 3. Discussion

The multifactorial nature of carotid atherosclerotic progression is illustrated by a recent case report describing the progression of initially non-obstructive carotid atherosclerosis to significant internal carotid artery stenosis despite sustained LDL cholesterol concentrations below recommended target levels under intensive medical therapy. The authors suggested that factors beyond conventional lipid control, including persistent low-grade inflammation, may contribute to disease progression. This observation further supports the concept that additional biological mechanisms may influence the development and progression of specific atherosclerotic phenotypes, including fibrocalcific plaque formation and arterial stenosis [13]. Over the years, cardiovascular disease progression and mortality have been found to be related to low levels of vitamin K, and therefore high levels of dp-ucMGP. Coronary heart disease and peripheral arterial disease risk were increased in a study group with elevated plasma dp-ucMGP concentration, independent of other cardiovascular risk factors [5]. MGP is an important local inhibitor of vascular calcification. Its biological activity depends on post-translational modification, including vitamin K-dependent γ-carboxylation and serine phosphorylation, with the inactive form, dp-ucMGP, converted to the fully active form, phosphorylated–carboxylated [14,15]. The dp-ucMGP represents the fully inactive form of the protein and has been used as a circulating biomarker of impaired MGP activation and poor vascular vitamin K status [15,16]. Elevated dp-ucMGP concentrations have been associated with vascular calcification in several clinical settings [6]. Therefore, the higher dp-ucMGP concentrations observed in patients with fibrocalcific plaques and/or arterial stenosis in the present study may reflect impaired activation of the MGP system and reduced calcification-inhibitory capacity in individuals with more advanced arterial disease.

The principal finding of this study was the association between dp-ucMGP and the presence of fibrocalcific plaques and/or arterial stenosis. Patients with fibrocalcific plaques and/or stenosis involving the carotid and/or arterial-limb territories had higher median dp-ucMGP concentrations than patients without fibrocalcific plaques or stenosis (1.29 vs. 1.07 ng/mL; *p* 0.047). Our findings are consistent with previous evidence linking circulating dp-ucMGP to vascular calcification burden. In a cohort of 128 peritoneal dialysis patients, serum dp-ucMGP levels rose progressively with the severity of abdominal aortic calcification and remained an independent predictor of calcification after multivariate adjustment [17]. These findings, together with our observation of higher dp-ucMGP concentrations in patients with fibrocalcific plaques and/or arterial stenosis, support the potential relevance of impaired MGP activation in the setting of established arterial calcific disease. Cahalane et al. investigated whether circulating dp-ucMGP could reflect differences in calcification patterns among patients with symptomatic atherosclerotic disease. In their cohort of patients undergoing carotid or peripheral lower-limb endarterectomy, peripheral arterial plaques showed a substantially greater calcification burden than carotid plaques, both in terms of calcified volume and, although not statistically significant, the number of calcified particles. Interestingly, circulating dp-ucMGP concentrations were significantly higher in patients with carotid disease than in those with peripheral lower-limb disease (*p* < 0.05) [7]. Patients with fibrocalcific plaques and/or arterial stenosis were significantly older than those in the control group [68.00 vs. 54.00, *p* < 0.001]. Although age has been reported to influence circulating MGP concentration, recent data suggest that the association between dp-ucMGP and cardiovascular outcomes may differ substantially across age groups. Younger patients with increased dp-ucMGP were associated with increased risk of cardiovascular events in contrast with older adults [18]. Moreover, Silaghi et al. reported higher serum total MGP concentration in healthy people younger than 40 years, suggesting that levels of t-MGP do not necessarily increase progressively with advancing age [19]. Therefore, the high dp-ucMGP concentration observed in patients with fibrocalcific plaques and/or arterial stenosis should not be interpreted only as an age effect, particularly given the specific vascular phenotype observed. In contrast, tMGP and Fetuin-A did not differ significantly between the two vascular groups (*p* 0.532 for tMGP and *p* 0.894 for Fetuin-A). The vascular phenotype was also associated with several metabolic and biochemical characteristics, including higher glucose, urea, creatinine, fibrinogen and uric acid concentrations, as well as differences in LDL and HDL cholesterol.

dp-ucMGP is known as the completely inactive form of MGP [6]. When assessing the relationship between estimated glomerular filtration rate and dp-ucMGP, the results showed that dp-ucMGP can serve as a marker of renal dysfunction: those with lower eGFR had higher dp-ucMGP levels [20]. The same authors studied histopathology by obtaining tissue from two healthy kidneys and four with renal dysfunction and found that those with calcium deposits had both carboxylated and uncarboxylated MGP, suggesting that MGP might also inhibit renal tissue calcification [21]. dp-ucMGP concentrations rise progressively with worsening renal function, with significantly higher levels observed in more advanced stages of chronic kidney disease, correlating with proteinuria, serum creatinine, and impaired glomerular filtration [22]. In the present study, dp-ucMGP demonstrated weak positive correlations with both urea and creatinine. Although the magnitude of these associations was modest, their direction is biologically plausible and consistent with previous evidence linking elevated dp-ucMGP concentrations with impaired renal function. In addition, renal dysfunction may influence circulating dp-ucMGP concentrations through altered metabolism or clearance. Therefore, the association between dp-ucMGP and renal function markers may partly reflect the close interaction between kidney dysfunction, impaired calcification inhibition, and vascular disease.

Although the kidneys represent an important component in the MGP pathway, growing evidence has also highlighted the relevance of MGP in cardiovascular biology, with studies of cardiac function providing significant insights into its potential clinical implications. MGP, uncarboxylated and unphosphorylated, was increased in patients with diastolic dysfunction, as measured by filling pressure echocardiographically using the E/e’ ratio [23]. In aortic stenosis, a higher plasma level of dp-uc MGP was found in those who were symptomatic. Even if the marker was not in accordance with the severity of aortic stenosis, the dp-ucMGP was increased in those with heart failure (assessed by the LVEF, CI, and NT-proBNP) and with long-term mortality [24]. In contrast to these findings, the ASTRONOMER trial concluded that an increased serum level of dp-ucMGP was an independent predictor of disease progression in aortic stenosis, but only in patients under 57 years old [25]. Increased levels of dp-ucMGP were linked to greater risks of both cardiovascular and overall mortality among stable individuals with established vascular disease. Specifically, patients whose dp-ucMGP concentrations were in the highest quartile (≥977 pmol/L) showed nearly a 90% higher risk of all-cause and cardiovascular death within five years, regardless of traditional cardiovascular risk factors [26]. Moreover, a different study found that not only are dp-ucMGP levels increased in people with acute coronary syndrome, but dp-ucMGP levels are also increased in people who present with non-ST-segment elevation myocardial infarction (NSTEMI), in contrast with those with ST-segment elevation myocardial infarction (STEMI), and dp-ucMGP serum levels also have a positive correlation with the GRACE score, a score used for assessing the risk of mortality [27]. In addition, Kumric et al. noticed that dp-ucMGP might be used as a marker for bleeding risk, based on patients who suffered a myocardial infarction and had increased dp-ucMGP levels [4]. Elevated plasma dp-ucMGP concentrations appeared to be linked to a lower likelihood of plaque hemorrhage. However, no significant relationships were identified between dp-ucMGP levels and either plaque lipid content or calcification. Similarly, plasma t-ucMGP concentrations showed no significant association with any of the assessed plaque characteristics [28].

In another Romanian cohort study, the authors investigated the relationship between MGP levels, risk factors for atherosclerosis, and carotid stenosis. They did not find any relationship between the marker and risk factors, except for cholesterol, but MGP was associated with the degrees of stenosis (insignificant/moderate/severe) based on the ultrasonography results. However, they measured the total desphospho-MGP (t-dpMGP) [29].

In diabetic patients, dp-uc MGP levels were in direct relationship with the risk of cardiovascular events, specifically with peripheral arterial disease and heart failure [30]. Another study on diabetic patients illustrated that serum levels of dp-uc MGP are useful for the determination of arterial stiffness via carotid-femoral pulse wave velocity [31]. Higher plasma dp-ucMGP concentrations were positively associated with several cardiovascular risk factors, including arterial stiffness (indicated by elevated estimated Pulse Wave Velocity), hypertension, history of cardiovascular disease (CVD), events, and obesity. These results suggest that dp-ucMGP may serve as a useful biomarker of cardiovascular risk [16].

Glucose concentrations were significantly higher in patients with fibrocalcific plaques and/or arterial stenosis than in the comparison group (*p* < 0.001), supporting the presence of a less favorable metabolic profile among patients with vascular disease. Hyperglycemia and diabetes are known to promote endothelial dysfunction, oxidative stress, inflammation, and vascular smooth muscle cell osteogenic transformation, thereby contributing to the development of vascular calcification and atherosclerotic progression. Therefore, the higher glucose concentrations observed in the study group may be relevant to the development of the fibrocalcific and stenotic vascular phenotype. However, despite this significant between-group difference, no statistically significant Spearman correlation was observed between glucose and dp-ucMGP concentrations. These findings are not contradictory because the two analyses address different questions. The group comparison indicates that higher glucose concentrations are associated with the presence of the fibrocalcific plaques and/or stenosis phenotype, whereas the correlation analysis evaluates whether increasing glucose concentrations are directly associated with increasing dp-ucMGP concentrations across individual patients. The absence of a significant correlation suggests that dp-ucMGP levels are not determined by glycemia alone and may instead reflect the combined influence of vascular calcification, MGP activation status, renal function, age, and other cardiovascular or metabolic factors.

In contrast to dp-ucMGP, tMGP concentrations were not significantly different between patients with and without fibrocalcific plaques and/or stenosis. This finding may indicate that the total circulating concentration of MGP does not adequately reflect its functional status. Because total MGP represents the overall amount of circulating protein rather than specifically identifying its biologically active or inactive forms, differences in MGP activation may not necessarily be reflected by differences in total MGP concentrations. Thus, the present findings support the potential relevance of assessing MGP status rather than tMGP concentration alone.

Taken together, the present findings suggest that dp-ucMGP may reflect a complex cardio-renal-metabolic environment rather than being determined by a single risk factor. Although patients with fibrocalcific plaques and/or stenosis had significantly higher dp-ucMGP concentrations, the correlation analysis demonstrated only weak associations with individual laboratory parameters. Specifically, dp-ucMGP was positively correlated with urea, creatinine, and triglycerides, whereas no significant correlation with glucose was observed. This pattern suggests that the association between dp-ucMGP and vascular calcification cannot be attributed solely to renal dysfunction, dyslipidemia, or contemporaneous glycemia. Instead, dp-ucMGP may integrate several processes related to vitamin K-dependent MGP activation, renal function, metabolic status, and vascular calcification.

Fetuin-A is a liver-produced glycoprotein released into the bloodstream, where it plays an important role in vascular disease associated with diabetes. In a prospective study, individuals with incident type 2 diabetes had lower pre-diagnosis Fetuin-A concentrations, which were associated with a significantly higher risk of cardiovascular complications, independent of established cardiometabolic risk factors. The concentration of Fetuin-A can be influenced by various disease and nutritional states, including chronic kidney disease, where impaired renal function and inflammation alter its regulation [32]. Elevated circulating Fetuin-A levels may contribute to insulin resistance by interfering with insulin receptor signaling through suppression of receptor tyrosine phosphorylation. As a result, persistent hyperglycemia and insulin resistance can disrupt normal endothelial and platelet function, fostering inflammation, narrowing of blood vessels, and increased clot formation. Together, these effects enhance the risk of atherosclerotic disease. Conversely, reduced Fetuin-A concentrations may promote vascular calcification, further aggravating the progression of atherosclerosis [26]. In a study on patients on hemodialysis, serum fibroblast growth factor 23 (FGF23), matrix Gla protein (MGP), and Fetuin-A were analyzed for their role in calcium-phosphate metabolism and for their predictive role in coronary artery calcification. FGF23 and Fetuin-A were found to be useful markers for coronary arterial calcification in patients on hemodialysis, with good sensitivity and specificity, and no correlation was found for overall serum MGP [33]. In addition, in calcific aortic valvular disease, Fetuin-A and Lipoprotein(a) were found to be elevated, while overall serum MGP levels were decreased [34]. More recent evidence from a meta-analysis including 3.208 patients with calcific aortic valve disease demonstrated lower circulating Fetuin-A concentration in patients with calcific aortic disease, supporting an association between reduced Fetuin-A levels and valvular calcification [35].

In a study involving the relationship between Fetuin-A and atherosclerotic plaques in the carotid and femoral arteries, the findings showed that lower blood levels of Fetuin-A were associated with a higher occurrence of calcified plaques detected by ultrasound. These results indicate that Fetuin-A may help protect against vascular calcification linked to atherosclerosis in patients with type 2 diabetes who do not have impaired kidney function [36]. Another study on the same vascular territories found that patients with at least one cardiovascular risk factor or even those with the diagnosis of cardiovascular disease, with increased plasma Fetuin-A levels had a slower atherosclerotic plaque progression in comparison with those with low levels of Fetuin-A [37]. In addition, Fetuin-A had reduced serum levels in patients with peripheral arterial disease, and it was more reduced in the cases with more severe lower extremity arterial calcification [38].

Fetuin-A is considered an important systemic inhibitor of ectopic calcification, but its circulating concentration may be influenced by multiple factors, including metabolic status, inflammation, liver function, renal function, and the presence of chronic disease. The absence of a significant association in the present study may indicate that circulating Fetuin-A does not discriminate between patients with and without fibrocalcific plaques and/or stenosis in this particular cohort. Alternatively, its role may be more complex and dependent on the specific vascular territory, stage of disease, or coexistence of other metabolic and inflammatory conditions.

Spearman correlation analysis identified several statistically significant associations; however, all correlation coefficients were weak in magnitude. dp-ucMGP showed weak positive correlations with urea, creatinine, and triglycerides. The associations with renal function markers are of particular interest because chronic kidney dysfunction is closely linked to disturbances in mineral metabolism and accelerated vascular calcification. Nevertheless, the low correlation coefficients indicate that renal function and triglyceride concentrations explain only a limited proportion of the variability in circulating dp-ucMGP levels. Similarly, tMGP showed only a weak positive correlation with fibrinogen, while Fetuin-A demonstrated weak associations with triglycerides. Overall, these findings suggest that circulating concentrations of the investigated biomarkers are influenced by multiple factors and cannot be explained by a single clinical or biochemical parameter.

Our study has some limitations. Firstly, it is across-sectional/observational design with a relatively small comparison group. Secondly, patients in the FCP+ group were significantly older than those in the comparison group. This difference is clinically relevant because aging is associated with the development and progression of atherosclerosis, vascular remodeling, and arterial calcification. Therefore, age may represent a factor in the observed relationship between dp-ucMGP and vascular disease. The higher dp-ucMGP concentrations observed in the study group should consequently be interpreted within the broader context of differences in age and cardiovascular risk profile between the two groups. Third, MGP serum levels might not reflect levels from all tissues and from the vascular lumen. Fourth, an important aspect of the present analysis is that the primary classification was based specifically on the presence of fibrocalcific plaques and/or arterial stenosis. This phenotype may represent a more advanced stage of vascular disease than the presence of atherosclerotic plaques alone. Therefore, the observed difference in dp-ucMGP may be particularly relevant with plaque calcification and vascular remodeling. The absence of significant differences in the other biomarkers further suggests that dp-ucMGP may provide information that is more closely related to this specific vascular calcification.

## 4. Materials and Methods

### 4.1. Study Design and Participant Recruitment

This observational study included 160 patients during a period of 5 months, evaluated for cardiovascular and heart disease. Demographic characteristics, cardiovascular risk factors, medical history, vascular findings, and laboratory parameters were collected for all participants. The study population included 87 women and 73 men, all Caucasian, who were referred to the Internal Medicine Clinic/Neurology and were admitted to Emergency Clinical Hospital Bihor and Clinical Hospital CF Oradea. Inclusion criteria were cardiovascular risk factors such as arterial hypertension, type 2 diabetes mellitus, dyslipidemia, hyperuricemia, overweight, ischemic heart disease, atrial fibrillation, or symptoms like dizziness and intermittent claudication. Participants were eligible for inclusion in the control and study groups if they provided written informed consent, had complete clinical and laboratory records, had undergone Doppler examinations, and yielded successful biochemical results.

**Figure 1:**
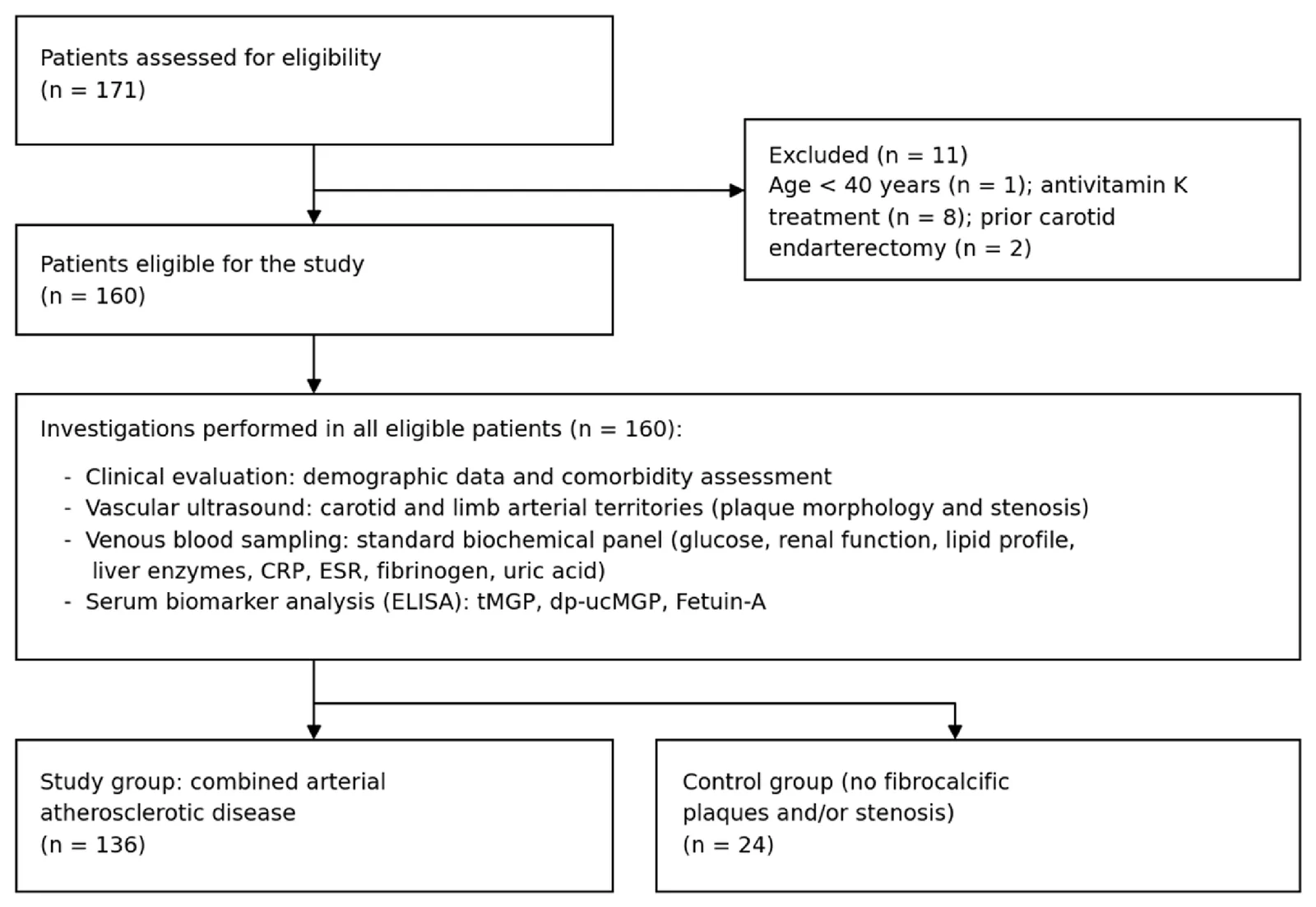
Study participant flow diagram.

Patients who underwent treatment with anti-vitamin K (coumarin anticoagulants) or treatment with vitamin K, and those who underwent carotid endarterectomy, were excluded from the study. Also, patients under 40 years were excluded. For the purposes of this study, arterial hypertension, dyslipidemia, obesity/overweight, and type 2 diabetes mellitus were defined according to established clinical criteria. Arterial hypertension was considered present in participants with a documented diagnosis of hypertension, current use of antihypertensive medication, or measured blood pressure ≥ 140/90 mmHg. Dyslipidemia was defined as the presence of at least one abnormal lipid parameter, including elevated total cholesterol, LDL cholesterol, or triglycerides or reduced HDL cholesterol, or the current use of lipid-lowering therapy. Body mass index (BMI) was calculated as weight in kilograms divided by height in meters squared (kg/m^2^). Participants were classified as underweight when BMI was <18.5 kg/m^2^, normal weight at 18.5–24.9 kg/m^2^, overweight at 25.0–29.9 kg/m^2^, and obese at ≥30.0 kg/m^2^. Type 2 diabetes mellitus was considered present in participants with a previously established diagnosis of type 2 diabetes, current use of glucose-lowering medication, or biochemical evidence fulfilling diagnostic criteria for diabetes (fasting plasma glucose ≥ 126 mg/dL [7.0 mmol/L], HbA1c ≥ 6.5% (Figure 1).

Of 171 patients initially assessed for eligibility, 11 were excluded (1 for age under 40 years, 8 for antivitamin K treatment, and 2 for prior carotid endarterectomy), leaving 160 patients eligible for the study. All eligible patients underwent a standardized clinical evaluation, vascular ultrasound of the carotid and limb arterial territories, venous blood sampling for a standard biochemical panel, and serum ELISA measurement of tMGP, dp-ucMGP, and Fetuin-A. Based on ultrasound findings, patients were then classified into the combined arterial atherosclerotic study group (n = 136) or the control group (n = 24).

**Figure 2:**
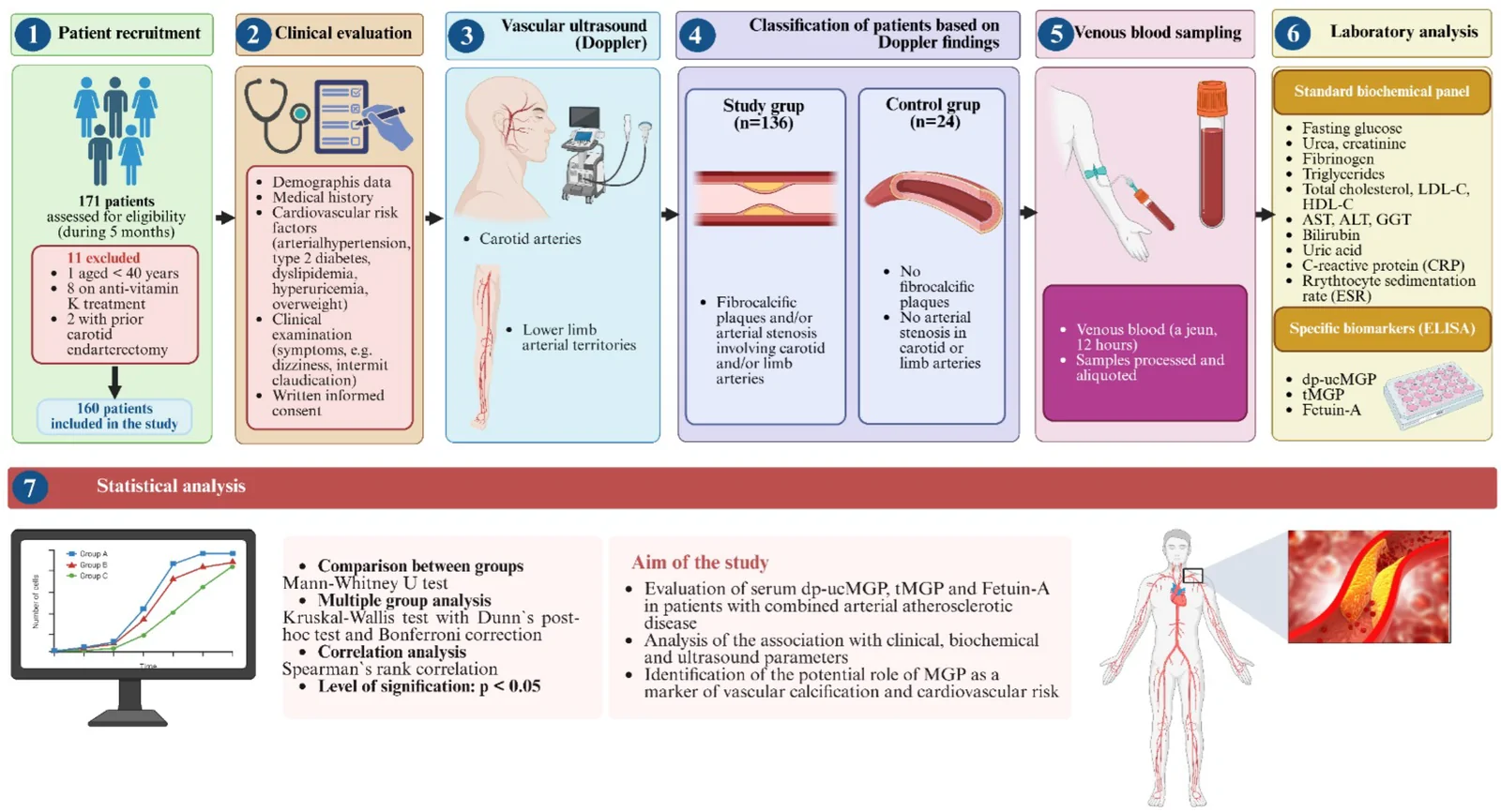
Experimental design. Overview of the study workflow: One hundred and sixty eligible patients underwent clinical evaluation and carotid/limb arterial ultrasound, and were classified into a combined arterial atherosclerotic study group (n = 136) and control group (n = 24). All patients underwent venous blood sampling for a standard biochemical panel and ELISA measurement of dp-ucMGP, tMGP, and Fetuin-A. Group comparisons and correlation analyses were performed using the Mann–Whitney U test, Spearman’s rank correlation, and the Kruskal–Wallis test with Dunn’s post hoc test and Bonferroni correction.

For the primary analysis, our study group was represented by patients with fibrocalcific plaques and/or arterial stenosis in the carotids and limbs together **(FCP+ group)** versus patients without stenosis or fibrocalcific plaques and with only fibroadipose plaques **(FCP− group)**, assessed by Doppler imaging. According to this classification, 136 patients were included in the FCP+ group, whereas 24 patients were included in the control group (FCP−). This classification was selected to identify patients with a more advanced structural vascular phenotype characterized by calcific atherosclerotic changes and/or hemodynamically relevant arterial narrowing (Figure 2).

### 4.2. Clinical and Biochemical Evaluation

Venous blood samples were collected in appropriate specimen tubes from all participants after an overnight fast of 12 h and analyzed for a standard biochemical panel comprising fasting glucose, urea, creatinine, fibrinogen, triglycerides, total cholesterol, LDL cholesterol, HDL cholesterol, liver enzymes (AST/GOT, ALT/GPT, GGT), total and direct bilirubin, uric acid, C-reactive protein (CRP), and erythrocyte sedimentation rate (ESR), using standard laboratory methods on Alinity c System using Abbott reagent kits (Abbott Laboratories, North Chicago, IL, USA) and erythrocyte sedimentation rate (ESR) (Alifax, Novaintermed, Voluntari, Romania).

### 4.3. Assessment of Serum dp-ucMGP, tMGP, and Fetuin-A

Venous blood samples were collected in citrated tubes and centrifuged. Serum was aliquoted into Eppendorf tubes and stored at −70 °C until further analysis. Serum concentrations of total matrix Gla protein (tMGP), dephosphorylated–uncarboxylated matrix Gla protein (dp-ucMGP), and Fetuin-A were measured using commercially available enzyme-linked immunosorbent assay (ELISA) kits. Dp-ucMGP concentrations were measured using an ELISA microplate reader (TECAN Sunrise™, Tecan Trading AG, Männedorf, Switzerland). Quantification of dephosphorylated uncarboxylated matrix Gla protein (dp-ucMGP) was performed using the Human MGP dp-ucMGP ELISA Kit from FineTest (Cat. No EH4755, Wuhan National Bio-industry Base, Wuhan, China). To measure total MGP, we used FineTest’s Human MGP (matrix Gla protein) ELISA Kit (Cat No EH1649, Wuhan National Bio-industry Base, China). For measuring Fetuin-A in serum we used the Elabscience Human FETUA (Fetuin-A) ELISA Kit (Cat No. E-EL-H0386, Wuhan National Bio-industry Base, China). Coefficients of variation were <10%.

### 4.4. Doppler Examination

B-mode ultrasound of the carotid arteries was performed using a Hitachi Arietta V60 ultrasound machine with a vascular transducer (4–12 MHz). For the examination, patients were placed supine with the head turned contralateral to the examination site. The classification of the patients was assessed by Doppler imaging (which visualizes the narrowing of the vessel lumen, disturbed flow, turbulence, and flow acceleration at the stenosis, and the type of the plaques) and by measurements of blood flow velocities within the artery. The peak systolic velocity (PSV) was measured in the carotid artery as well as the end-diastolic velocity (EDV). The same methods were applied for the inferior limb Doppler examination.

### 4.5. Objectives

The main objective of this study was to evaluate whether circulating levels of dephosphorylated–uncarboxylated matrix Gla protein (dp-ucMGP), total matrix Gla protein (tMGP), and Fetuin-A differ significantly between patients with and without combined arterial atherosclerotic disease, defined as the presence of fibrocalcific plaques and/or arterial stenosis involving the carotid and/or limb arterial territories. Secondly, we aimed to characterize the relationship between these three biomarkers and relevant clinical, metabolic, and vascular parameters (age, renal function, lipid profile, inflammatory markers) using correlation analysis and to determine whether dp-ucMGP, tMGP, and Fetuin-A levels differ across dyslipidemia and hyperuricemia status subgroups (present, under treatment, absent).

The board of Emergency Clinical Hospital Bihor and Clinical Hospital Căi Ferate Oradea approved the study. After their approval, we obtained the ethical committee’s acceptance of the University of Oradea (nr. 12527/30.07.2025). The study was performed in accordance with the fundamental principles of the Declaration of Helsinki.

### 4.6. Statistical Analysis

Vascular evaluation included assessment of carotid atherosclerosis and arterial stenosis. Carotid plaque morphology was categorized according to the presence of fibroadipose and fibrocalcific plaques. The presence or absence of carotid artery stenosis was recorded. Peripheral arterial involvement was also evaluated and included in the definition of combined arterial disease when fibrocalcific plaques and/or arterial stenosis were identified in the corresponding arterial territories. Our study group was represented by patients with fibrocalcific plaques and/or arterial stenosis in carotids and limbs together (n = 136) **(FCP+ group)** versus patients without stenosis or fibrocalcific plaques and with only fibroadipose plaques **(FCP− group)** (n = 24).

Continuous variables were assessed for distributional characteristics and were found to deviate from a normal distribution. Therefore, non-parametric statistical methods were used. Continuous variables were expressed as median and interquartile range, with the interquartile range calculated as the difference between the third and first quartiles. Categorical variables were presented as absolute numbers and percentages. For comparisons between two independent groups, the Mann–Whitney U test was used. This test was applied to compare dp-ucMGP, tMGP, Fetuin-A, and other continuous variables between patients with and without fibrocalcific plaques and/or arterial stenosis.

For comparisons involving three or more independent groups, the Kruskal–Wallis H test was used. When a statistically significant overall difference was identified, post hoc pairwise comparisons were performed using Dunn’s test with Bonferroni correction for multiple comparisons.

Spearman’s rank correlation coefficient (ρ) was used to assess associations between circulating concentrations of dp-ucMGP, tMGP, and Fetuin-A and selected continuous or ordinal clinical and laboratory variables. Correlation coefficients were interpreted primarily according to their magnitude and direction, and only statistically significant associations were highlighted in the main results. Correlation strength was interpreted as weak (|ρ| < 0.30), moderate (0.30–0.59), and strong (≥0.60). A two-sided *p*-value < 0.05 was considered statistically significant.

The statistical analysis was performed using RStudio Desktop (RStudio© PBC 2026.09.0-174, Boston, MA, USA).

## 5. Conclusions

In this cohort, higher circulating dp-ucMGP concentrations were associated with the presence of fibrocalcific plaques and/or arterial stenosis, whereas total MGP and Fetuin-A did not differ significantly between patients with and without vascular calcification. These findings suggest that the inactive form of MGP may provide information beyond total circulating MGP concentrations and may be more closely related to structural arterial changes characterized by calcification and stenosis.

Patients with fibrocalcific plaques and/or arterial stenosis were also older and exhibited a less favorable metabolic and renal profile, including higher glucose, urea, creatinine, uric acid, and fibrinogen concentrations. Although dp-ucMGP showed statistically significant correlations with urea, creatinine, and triglycerides, these associations were weak, indicating that its circulating concentration is likely influenced by multiple vascular, renal, and metabolic factors rather than by a single conventional cardiovascular risk factor.

Overall, our findings support the potential role of dp-ucMGP as a biomarker associated with fibrocalcific and stenotic arterial disease. However, because of the observational nature of the study, the relatively small comparison group, and potential confounding factors such as age and renal function, the results do not establish an independent or causal relationship. Larger prospective studies with multivariable adjustment are needed to determine whether dp-ucMGP provides independent predictive or prognostic value beyond established cardiovascular risk factors and whether it could contribute to improved identification and risk stratification of patients with advanced atherosclerotic vascular disease.

## Figures and Tables

**Table 1:** Baseline demographic, clinical, and biomarker characteristics of the overall study population (N = 160).

Variable	Value, n (%) or Median (IQR)
Age, years, median (IQR)	67.0 (IQR 13.00)
Aortic/abdominal atheromatosis, n (%)	74.00 (46.25)
Arterial hypertension, n (%)	141.00 (88.13)
Atrial fibrillation, n (%)	25.00 (15.63)
Chronic heart disease, n (%)	102.00 (64.15)
Chronic kidney disease, n (%)	47.00 (29.38)
Combined arterial atherosclerotic disease, n (%)	136.00 (85.00)
Creatinine, mg/dL—median (IQR)	0.90 (IQR 0.34)
CRP, mg/L—median (IQR)	2.80 (IQR 4.50)
Diabetes mellitus, n (%)	53.00 (33.13)
dp-ucMGP, ng/mL—median (IQR)	1.29 (IQR 0.84)
ESR, mm/h—median (IQR)	16.00 (IQR 20.00)
Female sex, n (%)	87.00 (54.38)
Fetuin-A, µg/mL—median (IQR)	388.50 (IQR 318.91)
Fibrinogen, mg/dL—median (IQR)	365.10 (IQR 121.21)
Glucose, mg/dL—median (IQR)	100.00 (IQR 29.00)
HDL-C, mg/dL—median (IQR)	52.05 (IQR 16.31)
LDL-C, mg/dL—median (IQR)	104.00 (IQR 65.00)
Obesity, n (%)	63.00 (39.38)
Stroke history, n (%)	22.00 (13.75)
tMGP, ng/mL—median (IQR)	15.79 (IQR 11.14)
Total cholesterol, mg/dL—median (IQR)	184.61 (IQR 74.69)
Triglycerides, mg/dL—median (IQR)	116.00 (IQR 81.15)
Urban residence, n (%)	95.00 (59.38)
Urea, mg/dL—median (IQR)	36.87(IQR 20.25)
Uric acid, mg/dL—median (IQR)	5.79 (IQR 1.93)

Values are n (%) for categorical variables and median (IQR) for continuous variables.

**Table 2:** Continuous clinical and laboratory variables by combined arterial atherosclerotic disease status (Mann–Whitney U test), ordered alphabetically.

Variable	FCP+ Group (n = 136)	FCP− Group (n = 24)	*p*-Value
Age, years	68.0	54.0	<0.0001
Creatinine, mg/dL	0.93	0.77	0.002
CRP, mg/L	2.90	1.80	0.052
D-bilirubin, mg/dL	0.14	0.11	0.233
dp-ucMGP, ng/mL	1.29	1.07	0.047
ESR, mm/h	16.00	14.00	0.103
Fetuin-A, µg/mL	388.50	403.05	0.894
Fibrinogen, mg/dL	374.43	330.05	0.046
GGT, U/L	29.60	23.35	0.189
Glucose, mg/dL	103.00	91.50	<0.0001
GOT, U/L	25.30	23.30	0.290
GPT, U/L	19.65	19.30	0.730
HDL-C, mg/dL	51.40	55.33	0.129
LDL-C, mg/dL	100.00	120.50	0.048
T-bilirubin, mg/dL	0.64	0.60	0.673
tMGP, ng/mL	15.79	12.67	0.532
Total cholesterol, mg/dL	177.00	213.28	0.065
Triglycerides, mg/dL	120.30	104.50	0.276
Urea, mg/dL	37.67	32.54	0.019
Uric acid, mg/dL	5.92	4.68	<0.0001

(FCP+): The presence of fibrocalcific plaques and/or arterial stenosis involving the carotid and/or limb arterial territories. (FCP−): The absence of fibrocalcific plaques or stenosis involving the carotid and/or limb arterial territories.

**Table 3:** Kruskal–Wallis test—clinical, laboratory, and biomarker variables across dyslipidemia subgroups.

Variable	Yes * n = 99	Under Treatment n = 49	No * n = 12	*p*-Value
Age, years	65.0	70.0	64.0	0.003
Creatinine, mg/dL	0.85	0.97	0.84	0.012
CRP, mg/L	3.05	2.10	4.20	0.518
D-bilirubin, mg/dL	0.11	0.16	0.15	0.006
dp-ucMGP, ng/mL	1.33	1.22	1.22	0.144
ESR, mm/h	16.00	18.00	14.00	0.517
Fetuin-A, µg/mL	424.85	336.85	336.52	0.430
Fibrinogen, mg/dL	362.91	369.72	378.84	0.855
GGT, U/L	26.60	32.05	36.85	0.442
Glucose, mg/dL	99.00	102.00	101.50	0.292
GOT, U/L	24.70	24.80	26.70	0.856
GPT, U/L	19.40	19.80	19.15	0.974
HDL-C, mg/dL	54.07	47.64	52.45	0.001
LDL-C, mg/dL	129.00	76.00	84.50	<0.0001
T-bilirubin, mg/dL	0.60	0.68	0.78	0.146
tMGP, ng/mL	16.05	14.94	12.46	0.143
Total cholesterol, mg/dL	212.38	148.54	162.82	<0.0001
Triglycerides, mg/dL	122.60	105.80	102.25	0.212
Urea, mg/dL	36.61	40.27	29.10	0.008
Uric acid, mg/dL	5.52	6.19	5.71	0.084

* Yes = dyslipidemia present (untreated); Under treatment = under pharmacological treatment for dyslipidemia; No = absent. Medians shown per subgroup; *p*-values from the Kruskal–Wallis omnibus test.

**Table 4:** Kruskal–Wallis test—clinical, laboratory, and biomarker variables across hyperuricemia subgroups.

Variable	YES n = 44	Under Treatment n = 7	No n = 109	*p*-Value
Age, years	69.5	73.0	64.0	0.005
Creatinine, mg/dL	0.98	1.10	0.85	<0.0001
CRP, mg/L	4.30	1.10	2.50	0.019
D-bilirubin, mg/dL	0.12	0.14	0.13	0.894
dp-ucMGP, ng/mL	1.40	1.25	1.26	0.517
ESR, mm/h	19.00	14.00	14.00	0.019
Fetuin-A, µg/mL	473.40	455.72	354.15	0.052
Fibrinogen, mg/dL	389.35	369.72	360.68	0.635
GGT, U/L	28.40	63.80	27.75	0.284
Glucose, mg/dL	106.50	95.00	99.00	0.190
GOT, U/L	25.25	34.30	23.70	0.065
GPT, U/L	21.65	32.60	19.00	0.072
HDL-C, mg/dL	47.23	49.02	53.59	0.085
LDL-C, mg/dL	97.00	55.00	113.50	0.014
T-bilirubin, mg/dL	0.69	0.64	0.61	0.345
tMGP, ng/mL	15.32	15.67	15.59	0.854
Total cholesterol, mg/dL	175.04	124.54	193.86	0.028
Triglycerides, mg/dL	148.60	113.00	105.95	<0.0001
Urea, mg/dL	49.00	43.47	32.40	<0.0001
Uric acid, mg/dL	7.39	5.98	5.28	<0.0001

Yes = dyslipidemia present (untreated); Under treatment = under pharmacological treatment for dyslipidemia; No = absent. Medians shown per subgroup; *p*-values from the Kruskal–Wallis omnibus test.

**Table 5:** dp-ucMGP, tMGP, and Fetuin-A by combined arterial atherosclerotic disease status (Mann–Whitney U test).

Biomarker	FCP + Group	FCP− Group	*p*-Value
dp-ucMGP, ng/mL	1.29 (0.99–1.82)	1.07 (0.91–1.37)	0.047
Fetuin-A, µg/mL	388.50 (248.45–613.41)	403.05 (305.93–484.83)	0.894
tMGP, ng/mL	15.79 (9.68–20.97)	12.67 (9.55–19.00)	0.532

**Table 6:** Spearman correlations between dp-ucMGP, tMGP, and Fetuin-A and clinical/vascular variables.

Biomarker	Correlated Variable	ρ	*p*
dp-ucMGP	Urea	0.175	0.034
dp-ucMGP	Creatinine	0.183	0.026
dp-ucMGP	Triglycerides	0.197	0.017
Fetuin-A	Triglycerides	0.176	0.029
tMGP	dp-ucMGP	0.443	0.001
tMGP	Fibrinogen	0.208	0.014

ρ = Spearman correlation coefficient. Only significant pairs (*p* < 0.05) are present in the table.

## Data Availability

The raw data involved in this study can be obtained upon reasonable request addressed to Teodora Bianca Tirla-Sirca (sirca.teodorabianca@uoradea.ro).
